# From Genes to Milk: Genomic Organization and Epigenetic Regulation of the Mammary Transcriptome

**DOI:** 10.1371/journal.pone.0075030

**Published:** 2013-09-26

**Authors:** Danielle G. Lemay, Katherine S. Pollard, William F. Martin, Courtneay Freeman Zadrowski, Joseph Hernandez, Ian Korf, J. Bruce German, Monique Rijnkels

**Affiliations:** 1 Genome Center, University of California Davis, Davis, California, United States of America; 2 Department of Food Science and Technology, University of California Davis, Davis, California, United States of America; 3 Gladstone Institutes, Institute for Human Genetics, and Department of Epidemiology and Biostatistics, University of California San Francisco, San Francisco, California, United States of America; 4 USDA/ARS Children's Nutrition Research Center, Department of Pediatrics, Baylor College of Medicine, Houston, Texas, United States of America; Harvard School of Public Health, United States of America

## Abstract

Even in genomes lacking operons, a gene's position in the genome influences its potential for expression. The mechanisms by which adjacent genes are co-expressed are still not completely understood. Using lactation and the mammary gland as a model system, we explore the hypothesis that chromatin state contributes to the co-regulation of gene neighborhoods. The mammary gland represents a unique evolutionary model, due to its recent appearance, in the context of vertebrate genomes. An understanding of how the mammary gland is regulated to produce milk is also of biomedical and agricultural importance for human lactation and dairying. Here, we integrate epigenomic and transcriptomic data to develop a comprehensive regulatory model. Neighborhoods of mammary-expressed genes were determined using expression data derived from pregnant and lactating mice and a neighborhood scoring tool, G-NEST. Regions of open and closed chromatin were identified by ChIP-Seq of histone modifications H3K36me3, H3K4me2, and H3K27me3 in the mouse mammary gland and liver tissue during lactation. We found that neighborhoods of genes in regions of uniquely active chromatin in the lactating mammary gland, compared with liver tissue, were extremely rare. Rather, genes in most neighborhoods were suppressed during lactation as reflected in their expression levels and their location in regions of silenced chromatin. Chromatin silencing was largely shared between the liver and mammary gland during lactation, and what distinguished the mammary gland was mainly a small tissue-specific repertoire of isolated, expressed genes. These findings suggest that an advantage of the neighborhood organization is in the collective repression of groups of genes via a shared mechanism of chromatin repression. Genes essential to the mammary gland's uniqueness are isolated from neighbors, and likely have less tolerance for variation in expression, properties they share with genes responsible for an organism's survival.

## Introduction

Bacterial operons exemplify how a gene's expression is affected by proximity to neighboring genes. When a gene translocates from one genomic position to another, the expression of that gene often changes. Likewise in mammalian genomes, the alteration of a gene's neighborhood over evolutionary time can alter gene expression [1], [2]. Essential genes – those required for an organism's survival – are more resistant to altered gene expression that results from genomic rearrangement [1]. Neighborhoods of mammalian co-expressed genes often form through tandem duplications and are preferentially maintained when they are composed of functionally linked, non-essential genes [3]. However, the mechanisms by which neighboring genes are co-expressed in eukaryotic genomes are incompletely understood.

The chromatin configuration surrounding a gene – its epigenetic state – also influences the capacity of that gene to be expressed. Nuclear DNA is packaged into chromatin; this organizes the genome into regions that are more or less accessible to the transcription machinery. The cumulative epigenetic state of all genes in a cell determines the cell's expression capacity and is associated with its differentiation state and cell identity [4], [5]. We hypothesized that the epigenetic state contributes to the co-regulation of gene neighborhoods.

We explore this hypothesis using milk production and the mammary gland as a model system. Beyond the specific applications of human lactation and dairy science, the mammary gland represents a unique evolutionary model. By 500 million years ago, after the Cambrian explosion, animals had obtained most of the modern features present today, while the mammary gland evolved ∼350 million years later and represents a fairly recent adaptation. Our goal, in part, was to determine the extent to which clustering of mammary genes into neighborhoods facilitated the evolution of milk production. In the absence of rich transcriptional data for milk production across many species, we used comparative genomics paired with transcriptional data from one model species to find conserved gene neighborhoods. Given that the casein gene neighborhood arose in mammals to produce important milk proteins, we expected that genes in other conserved neighborhoods would also be important for milk production.

In a previous study, we observed that a subset of non-redundant expressed sequence tags derived from bovine mammary gland tissue were co-located in the bovine genome statistically more frequently than expected by chance [3]. Although this study provided some preliminary evidence that genes expressed in the mammary gland do form gene neighborhoods, we did not compare neighborhood occurrence to other tissues. More importantly, neighborhood identification was limited by arbitrary constraints (e.g. minimum of 3 genes, window of 500 kilobase pairs) and did not incorporate evolutionary conservation across other mammalian genomes. To address these problems, we developed a new bioinformatics tool, called Gene Neighborhood Scoring Tool (G-NEST) [3], and applied it to gene expression data from mouse mammary glands.

To interpret mammary gene neighborhoods in the context of chromatin, we conducted Chromatin Immuno-precipitation (ChIP)-Seq on mammary and liver tissues of lactating mice for three histone modifications: H3K36me3, H3K4me2, and H3K27me3. H3K4me2 and H3K36me3 enrichment are associated with open and actively transcribed genes, whereas H3K27me3 is associated with closed, transcriptionally inactive chromatin [5]–[7]. In this manuscript, we report the genomic organization and chromatin state of genes expressed in lactating mammary tissue.

## Methods

### Ethics statement

ICR mice were obtained from Harlan laboratories and housed in an American Association of Laboratory Animal Care-accredited facility at Baylor College of Medicine following guidelines outlined by the institutional Animal Care and Use Committee, approved protocol AN-3455.

### Gene expression data selection and pre-processing

The “Atlas” data set, which contains gene expression intensity estimates from two replicates of each of 61 mouse tissues [8], was downloaded from NCBI GEO [9]. One of these tissues is the mouse mammary gland harvested during lactation. The “Mammary” data set consists of 40 Affymetrix microarrays: 10 time points, each with 4 biological replicates of the mouse mammary gland, as described previously [10]. The time points span the lactation cycle from early pregnancy through involution in FVB mice. This dataset is available at NCBI GEO [9].

Each probe on the Affymetrix chip was remapped to an Ensembl transcript using methods described by Dai et al. [11]. Genome locations for these transcripts were downloaded from the Ensembl database, release 52 [12]. Genome coordinates for NCBI reference sequences and all mRNA for mouse genome version mm9 were obtained using the UCSC Table Browser. Probes on the microarray for which there were not at least 5 “Present” MAS5 detection calls were removed because at least 5 values are needed for the correlation function. The transcripts associated with the remaining probes were ordered according to genome location. Overlapping transcripts were handled as described previously [3].

Gene expression values were obtained by pre-processing the data sets using the customized pre-processing algorithms identified by Harr and Schlotterer [13], which generated the highest correlation coefficient known bacterial operons. These pre-processing algorithms, in R/Bioconductor [14], include background correction “mas,” normalization algorithm “invariantset,” perfect match correction algorithm “mas,” and summary algorithm “liwong.” All expression values were log transformed (base 2).

### Identification of gene neighborhoods

To identify gene neighborhoods based on the “Mammary” data set, we used the Gene Neighborhood Scoring Tool (G-NEST) [3], with a minimum and maximum gene count of 2 and 10, respectively. Syntenic blocks for G-NEST were generated using Cinteny [15], the parameters minBlk, maxGap, and numMark set to 100 kb, 1 Mb, and 2, respectively. Single copy (1∶1) orthologs from Ensembl Genes 62 were uploaded to Cinteny to generate syntenic blocks for the following genomes relative to the mouse genome assembly NCBIM37, also known as mm9: human (*Homo sapiens*) GRCh37.p3, chimpanzee (*Pan troglodytes*) CHIMP2.1, gorilla (*Gorilla gorilla*) gorGor3, orangutan (*Pongo abelii*) PPYG2, macaque (*Macaca mulatta*) MMUL_1.0, marmoset (*Callithrix jacchus*), mouse (*Mus musculus*) NCBIM37, rat (*Rattus norvegicus*) RGSC3.4, cow (*Bos taurus*) Btau_4.0, horse (*Equus caballus*) EquCab2, and dog (*Canis familiaris*) CanFam_2.0.

For each putative neighborhood, G-NEST combines gene expression and synteny information to determine a Total Neighborhood Score (TNS) indicating to what extent the putative cluster of genes is a “neighborhood.” The TNS is a score from 0 (not a neighborhood) to 1 (neighborhood). It is defined as follows: TNS  =  (SS) (ANC) for p≤0.05 else 0, where SS (Synteny Score) is the proportion of genomes in which synteny is maintained, ANC (Average Neighborhood Correlation) is the average of all pairwise correlations of all genes in the neighborhood, and p is the p-value computed from randomized transcriptomes (i.e. the probability that the ANC is observed by chance).

### ChIP-Seq data generation and analysis

To identify actively transcribed genes/genomic regions and regions in the genome that have been silenced, we performed ChIP-seq using antibodies against histone H3-di-methylated-lysine 4 (H3K4me2) and histone H3-tri-methylated-lysine36 (H3K36me3), both associated with actively transcribed genes, as well as histone H3-tri-methylated-lysine27 (H3K27me3), associated with silenced genes and genomic regions [5]–[7]. ChIP-Seq data for histone mark H3K4me2 were generated previously [16] and are available in GEO: GSE25105. ChIP-Seq data for histone marks H3K36me2 and H3K27me3 were generated using the same methods as described for H3K4me2 [16] using pooled mammary gland or liver tissue from 4–6 ICR mice at lactation day 8. ICR mice were obtained from Harlan laboratories and housed in an American Association of Laboratory Animal Care-accredited facility at Baylor College of Medicine following guidelines outlined by the institutional Animal Care and Use Committee, approved protocol AN-3455. Raw reads generated from Illumina/Solexa GAII were mapped to mouse reference genome (NCBI37/mm9) using Eland (Illumina) with maximally 2 mismatches tolerated. These two new data sets have been deposited in NCBI's GEO database: GSE25131.

Most peak calling algorithms for ChIP-seq data are designed with narrow factor occupancy in mind (e.g. transcription factor binding sites). However, histone modifications like H3K36me3 and H3K27me3 usually have a more broad distribution in the genome, spanning larger regions (genes, K36me3; genomic regions, K27me3). To identify enrichment of more diffuse/broad histone modification signals over larger regions, we applied the SICER algorithm [17] to the genome-wide raw sequence reads of H3K4me2, H3K36me3, and H3K27me3 occupation sites in the lactating mammary gland and in liver as described previously [16]. Input (unenriched)–seq read libraries were used as a control in both analyses. SICER's default parameters were used except for the change of species to mm9 and the gap size. The window size was kept at 200 bp because this is approximately the length of a nucleosome plus linker. The gap size parameter is a multiple of the window size, but the optimal choice of this parameter depends on the characteristics of the chromatin modification. To determine an appropriate gap size, SICER was iteratively run with increasing gap size and the aggregate island score was plotted as a function of gap multiple to find the gap size for which the maximum is reached. Optimal gap sizes of 400 bp, 1200 bp, and 20 kb were chosen for H3K4me2, H3K36me3, and H3K27me3, respectively.

### Chromatin Domain Scores

For each gene, we used the SICER peaks of the three marks in the two tissues to compute a mammary-to-liver Chromatin Active Domain Ratio (CADR) and Chromatin Silenced Domain Ratio (CSDR). The mammary-to-liver CADR is the sum of the mammary K4 and K36 peaks across a genomic region divided by the sum of the liver K4 and K36 peaks across the same genomic region (when summing several SICER peaks, each peak's contribution to the sum is equal to its height times its width). The mammary-to-liver CSDR is the sum of the mammary K27 peaks divided by the sum of the liver K27 peaks (Figure S1). For each gene, CADR and CSDR scores were computed using the genomic region from transcription start to transcription end.

The Neighborhood Chromatin Active Domain Ratio (NCADR) was computed in the same manner as the CADR, except that the start end and end points of the genomic region were the start and end points of the gene neighborhood, rather than of the transcription start and end of a single gene. Likewise, the Neighborhood Chromatin Silenced Domain Ratio (NCSDR) was computed in the same manner as the CSDR, with the neighborhood as the genomic region.

We additionally defined a chromatin domain score (DS) that incorporated all three histone marks. The DS is defined as follows: DS  =  log (CADR +1) – log (CSDR +1). If a gene is associated with a positive DS, this indicates more active and/or less silenced chromatin in the mammary relative to liver tissue. Negative DSs indicate less active and/or more silenced chromatin in the mammary gland relative to liver tissue. Scores near zero indicate very similar chromatin states in the mammary gland compared with liver tissue.

### Statistical analyses

A Wilcoxon rank sum test with continuity correction (also known as a Mann-Whitney U) from the R programming language was used to determine if the mean of the distribution differed between gene sets of interest (e.g. high expressing vs low expressing genes). A two-sample Kolmogorov-Smirnov test was used to determine if the observations associated with a gene set were drawn from the same distribution as another gene set. For both statistical tests, significance was determined by a p-value ≤0.05.

## Results

### Mammary gene co-expression is correlated with genomic distance

To verify whether relative gene position has any influence on co-expression of genes in the mammary gland, we computed the mean pairwise gene expression correlation (Spearman's) for genes within a given genomic interval. We included all non-overlapping transcripts probed in the Mammary data set (Methods). Each pairwise correlation was categorized as a different-chromosome correlation if the genes were on different chromosomes or by genomic distance if the genes were on the same chromosome. The smallest mouse chromosome in the mm9 assembly is approximately 61 megabases (Mb) so statistics were collected at 10 kilobase (Kb) increments up to 60 Mb on the same chromosome. For example, if the start sites of two genes were 351 Kb apart, their pairwise gene expression correlation contributed to the mean in the 350–360 Kb interval. The different-chromosome mean gene expression correlation was computed for all pairwise combinations of genes on different chromosomes.

**Figure 1:**
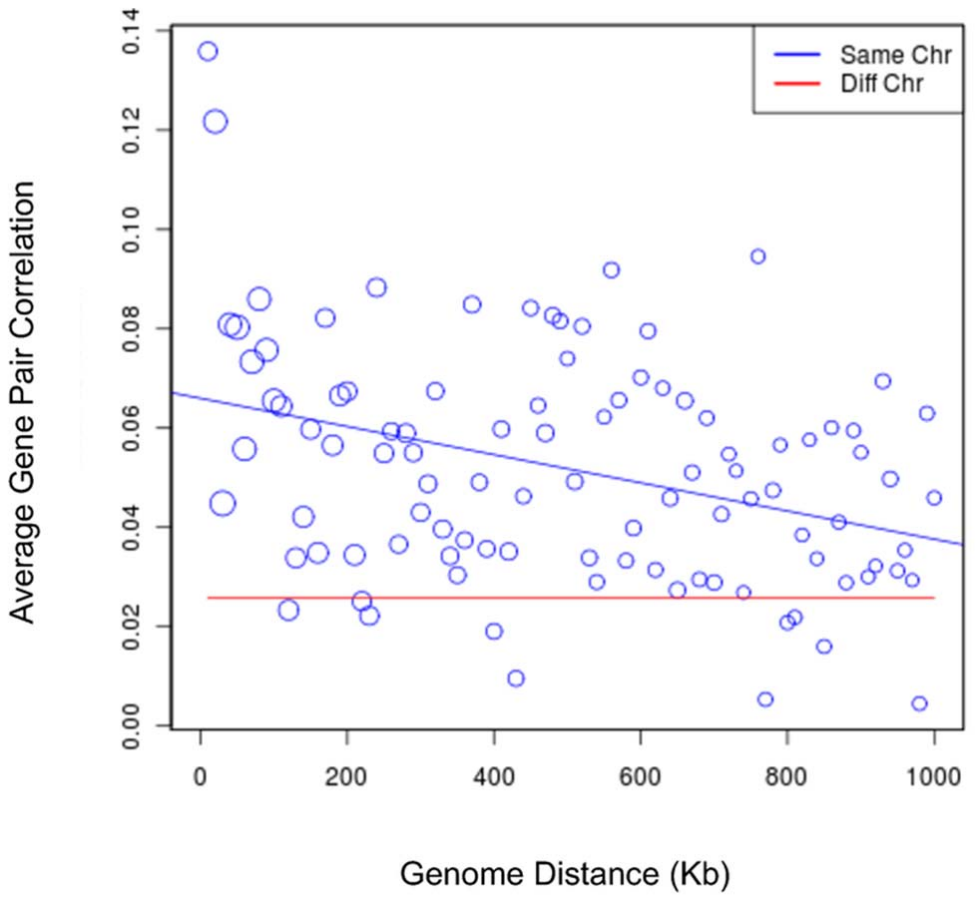
Correlation of mammary gene expression profiles with genomic distance. The *x*-axis is genomic distance in Kb. The *y*-axis is average correlation. Each circle represents the mean correlation of all gene pairs within that genomic interval on the same chromosome. The size of the circle indicates how many gene pairs were used for that data point. The red line indicates the mean correlation of gene pairs on different chromosomes.

As expected based on prior experiments with other tissues [18], the correlation of gene expression decreased with increasing genomic distance (Figure 1). In other words, nearby genes had better correlated expression than those further away. The effects of genomic distance were apparent even with genes up to 1Mb apart. We conducted an identical analysis using all tissues of the Atlas data set [8] and found a similar trend (Figure S2). The correlations, while weak, observed in the mammary gland data were similar to those of all tissues (x-axis intercepts at approximately 0.07 and 0.05, respectively). These data confirmed an influence of genomic distance on gene expression in the mammary gland similar to other tissues.

### Mammary-expressed genes are organized into neighborhoods that are shared with other tissues

**Figure 2:**
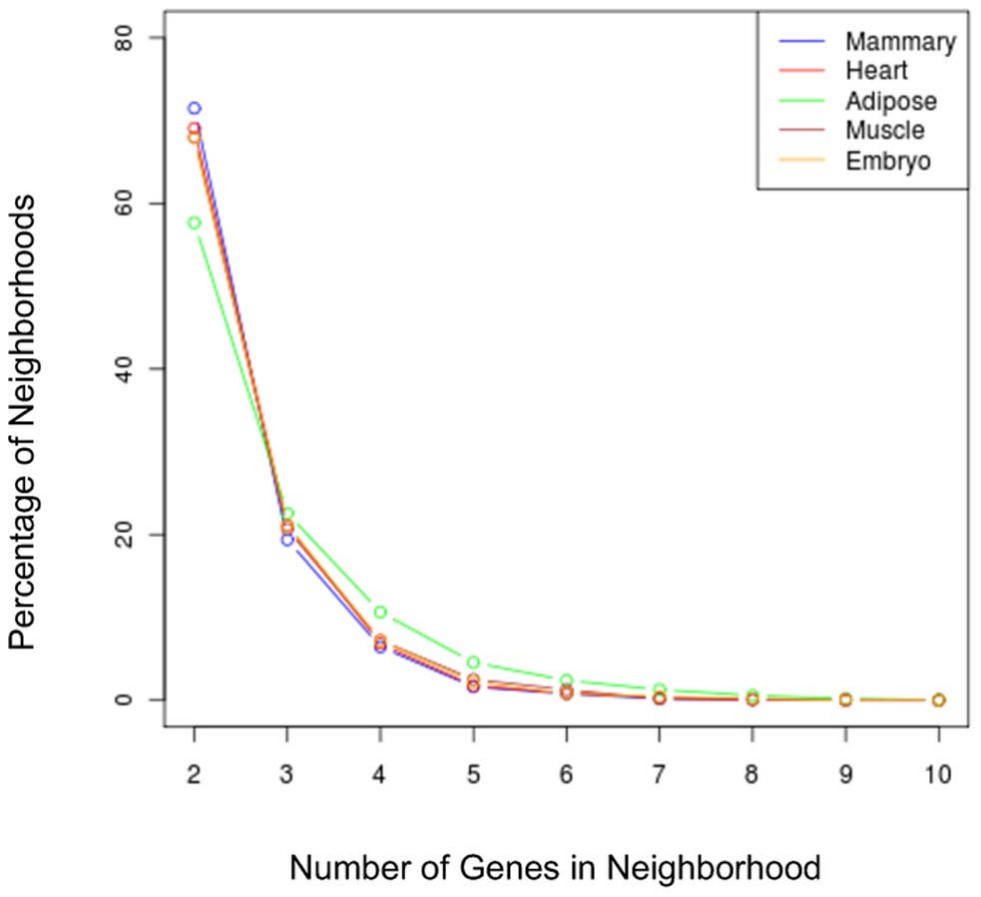
Comparison of mammary gene neighborhood size with those of other tissues. The *x*-axis is the number of genes in the neighborhood (defined here as adjacent genes co-expressed in the given tissue) and the *y*-axis is the relative percentage of neighborhoods with this size.

For direct comparison of the mammary gland with other tissues, we used the genome-wide “Atlas” mouse gene expression data from 61 tissues [8] that included two replicates of the lactating mammary gland. Due to the limited number of replicates per tissue, all cross-tissue analyses of gene neighborhoods utilized a simplistic neighborhood definition: adjacent genes whose transcripts are “Present” in both replicates of the tissue. Using this definition, we asked whether there were more gene neighborhoods in the lactating mammary gland than expected. Significantly more mammary-expressed genes occurred in neighborhoods than expected by chance (p<0.05). Likewise, there were fewer genes “isolated” (expressed, but adjacent to non-expressed genes) in the mammary gland than expected by chance (p<0.05). Neighborhood sizes ranged from 2–5 genes with a median size of 97 Kb and (5th to 95th percentiles of 4 to 963 Kb). Using the Atlas data, the sizes of mammary gene neighborhoods were not significantly different from other tissues in terms of number of genes (Figure 2) or length in base pairs (Figure S3).

**Figure 3:**
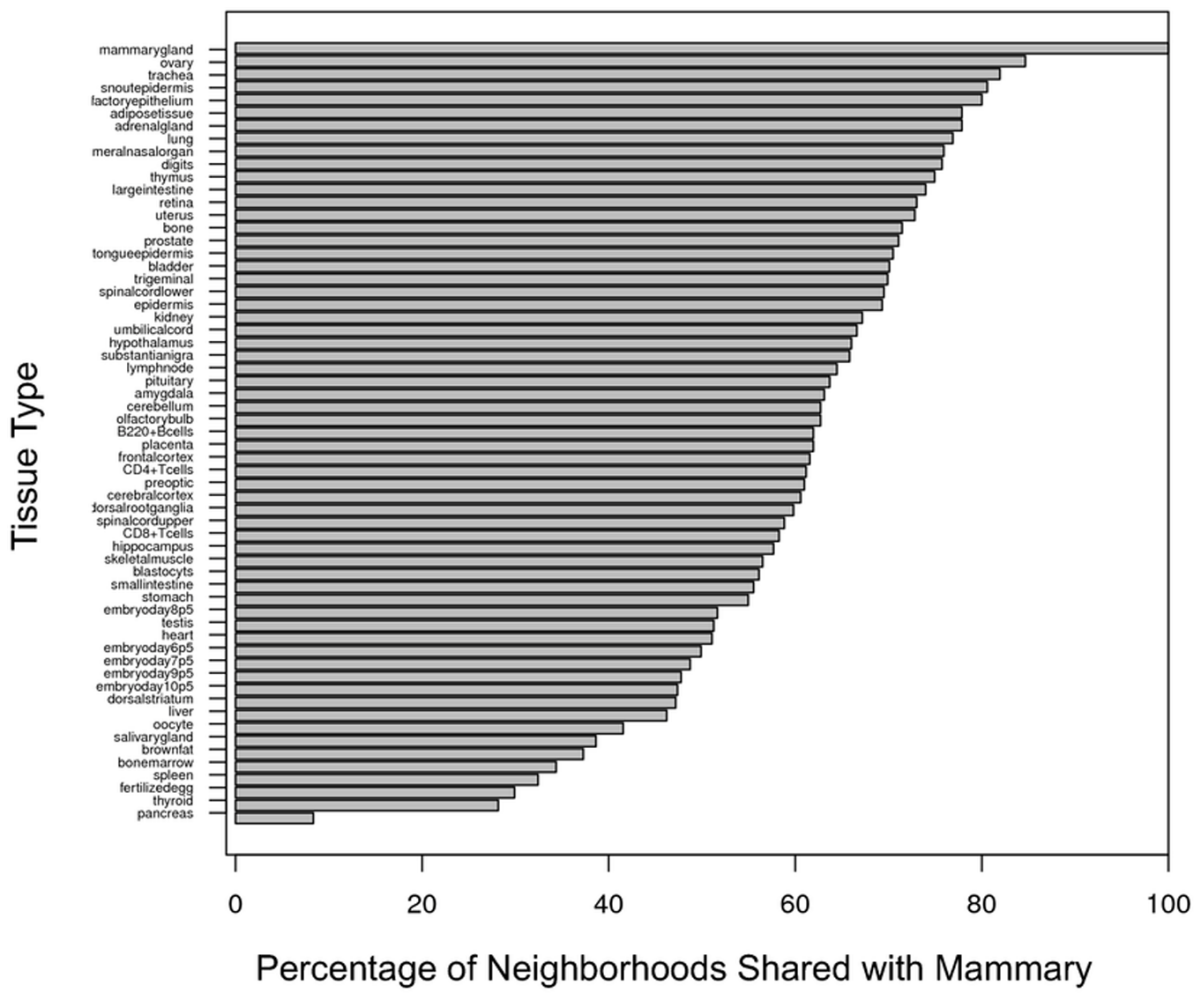
Percentage of other-tissue neighborhoods shared with mammary gland. The *y*-axis lists the probed tissues. The *x*-axis denotes the percentage of mammary gene neighborhoods that are shared with each probed tissue. The tissues were ranked based on the percentage of shared neighborhoods.

We also investigated to what extent mammary gene neighborhoods were shared with other tissues. A mammary gene neighborhood was deemed “shared” with another tissue if transcripts of all genes in the putative neighborhood were also detectable in the second tissue (Figure 3). Because the mammary gland is made up of epithelial and adipose tissue, with more epithelial than adipose at the time of lactation in mice, we expected these tissue types or other tissues made primarily of similar cells to share the most neighborhoods. Indeed, many of the tissues with the highest percentage of gene neighborhoods shared with the mammary gland – trachea (81.9%), snout epidermis (80.6%), medial olfactory epithelium (80.0%) – were dominated by epithelial cells. However, the tissue that shared the most gene neighborhoods with the lactating mammary gland was the ovary (84.7% shared). This is interesting because both the mammary gland and the ovary are regulated in response to hormones such as estrogen, progesterone, or prolactin.

That nearly 85% of mammary gene neighborhoods were shared with the ovary suggested that as much as 15% of neighborhoods in the mammary gland may be unique. Surprisingly, not a single gene neighborhood appeared to be entirely unique to the mammary gland. Even the well-known casein neighborhood was expressed, albeit at much lower levels, in other tissues.

### Histone marks specified both active and silenced genes during lactation

As shown in Figure 3, mammary and liver tissues were among the most divergent in terms of shared neighborhoods of co-expressed genes. The organization of the chromatin a gene resides in – its epigenetic state – has a direct influence on the capacity of that gene to be expressed. To examine how epigenetic states as represented here by specific patterns/signatures of post-translational modifications on histones correlated with expression of genes, we determined epigenetic states of genes unique to the mammary gland or liver. We conducted Chromatin Immuno- precipitation (ChIP)-Seq on mammary and liver tissues of lactating mice for three histone modifications: H3K36me3, H3K4me2, and H3K27me3. H3K4me2 and H3K36me3 enrichment are associated with open and actively transcribed genes, while H3K27me3 is associated with closed, transcriptionally inactive, chromatin [5]–[7].

To determine whether these epigenetic marks were consistent with gene expression, genes were classified as mammary-expressed or liver-expressed using the “Atlas” data set and as epigenetically “active” or “silenced” by scoring enrichment of histone marks (Methods). High mammary-to-liver Chromatin Active Domain Ratio (CADR) scores for each gene, based on the histone marks associated with open chromatin (H3K36me3 and H3K4me2) in that gene's region, were indicative of more “active chromatin” in the lactating mammary gland relative to the liver surrounding that gene's DNA. Likewise, high mammary-to-liver Chromatin Silent Domain Ratio (CSDR) scores for each gene, based on the histone mark associated with closed chromatin (H3K27me3), should be indicative of more “silenced chromatin” in the mammary relative to the liver in that gene's region. The CADR and CSDR are both required; gene regions without active histone marks are not necessarily silenced and gene regions without silencing histone marks are not necessarily active. However, a liver-to-mammary version of these ratios would be redundant because they are merely the inverse of the mammary-to-liver CADR and CSDR.

**Figure 4:**
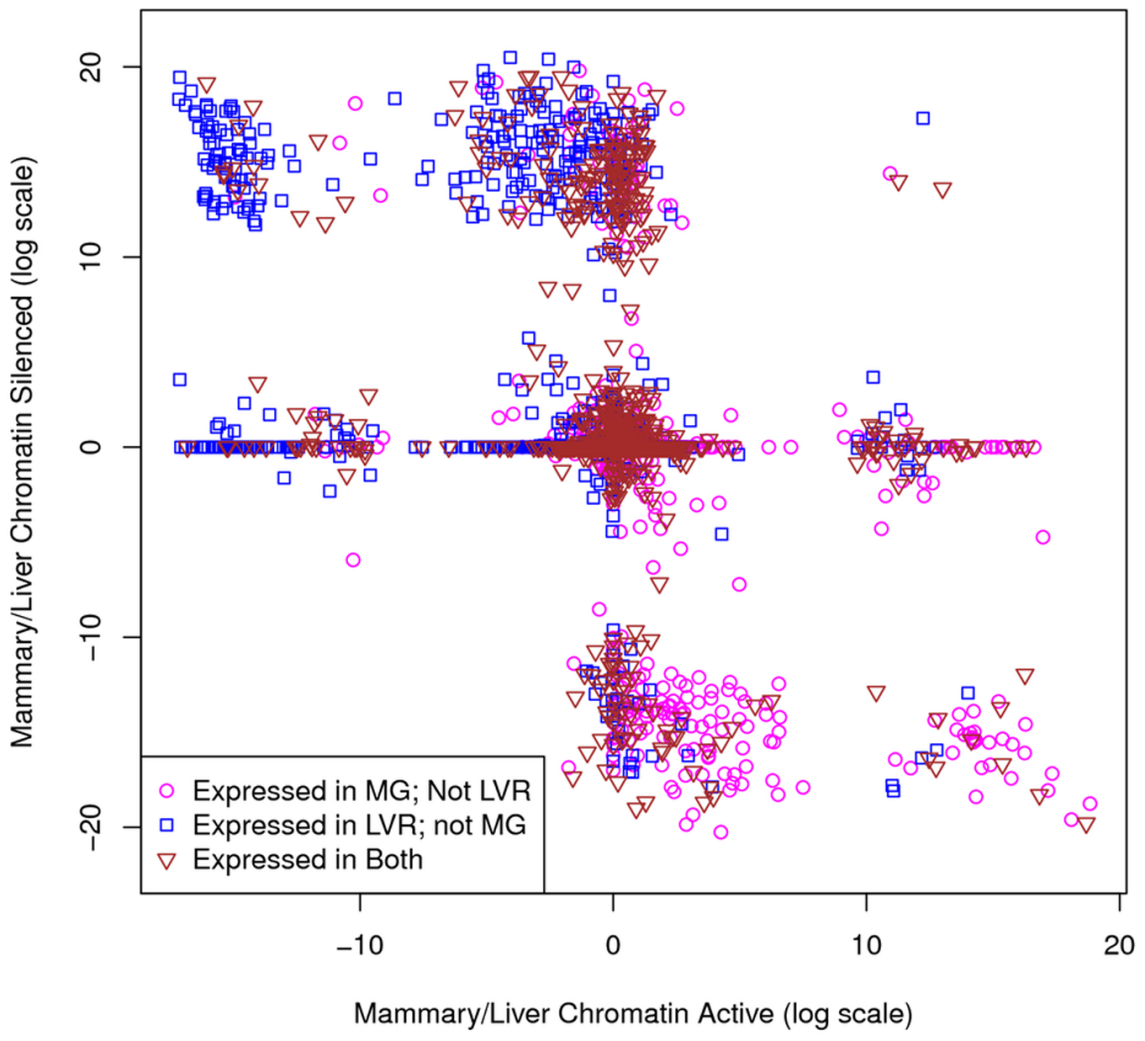
Intersection of chromatin domain scores with gene expression. The expression of each gene was determined using the “lactating mammary gland” and “liver” tissue replicates from the Atlas data set (Methods). Mammary-to-liver CADR and CSDRs were determined using histone marks H3K4me, H3K36me3, and H3K27me3 enriched in ChIP-Seq of mammary and liver tissues (Methods).

Mammary-to-liver CADR and CSDR scores were computed for each gene, encompassing the genomic region from transcription start to transcription end. CADR and CSDR scores were then log-transformed, plotted, and annotated by mammary and liver expression (Figure 4.) First, it is interesting that there were no genes in the bottom left tertile (mammary not silenced and not active) and only four genes in the upper right tertile (mammary active and silenced), suggesting that the epigenetic marks were remarkably self-consistent. Second, most genes fell on one or both of the two major axis (CADR  = 0 or CSDR  = 0), suggesting that most genes were equally active or silenced in the mammary gland and in liver tissue. The remaining areas denote genes that were uniquely active/not silenced in the mammary gland (bottom right) and uniquely silenced/not active in the mammary gland (top left).

To determine the function of genes that were uniquely active or silenced in the lactating mammary gland, we conducted functional enrichment analyses of the highest-scoring genes (active, log (CADR) >8; silenced, log (CSDR) ≤8; lower right corner of Figure 4). Functional clustering analysis of these 82 genes uniquely active in the lactating mammary gland compared with liver yielded just one significant cluster: glycoprotein/disulfide bond/signal peptide/secreted. A manual review of these genes suggests they are primarily involved in synthesizing and secreting products, immune defense, or maintenance of mammary gland structure.

Functional clustering analysis of the 130 genes uniquely silenced in the lactating mammary gland relative to the liver (log (CADR) ≤8; log (CSDR) >8; upper left corner of Figure 4) also yielded “secretion” as a significant function. However, additional significant clusters confirmed that the secretory products were liver-specific, such as chylomicrons, high-density lipoproteins, etc. The remaining significant clusters were associated with known functions of the liver: drug metabolism, blood coagulation, and acute inflammatory response. Together, these analyses suggest that epigenetic marks highlight genes that are uniquely active or silenced in the mammary gland, relative to the liver, with functions consistent with known biology.

### Chromatin and gene expression status are similar between tissues

The clustering of data points along the axes in Figure 4 suggested that, for most genes, the chromatin state was shared between mammary and liver tissues. To quantify the degree of shared chromatin, we computed a Domain Score (DS) that incorporated all three histone marks (Methods). Genes with a positive DS had more active and/or less silenced chromatin in the mammary relative to liver tissue. A negative DS was indicative of less active and/or more silenced chromatin in the mammary gland relative to liver tissue. Scores near zero indicated very similar chromatin states in the mammary gland compared with liver tissue. For mammary-to-liver comparisons, DS ranged from –20.7 to +19.0. More than 82% of all genes had a DS between –2 and +2, confirming that, for most genes, chromatin state was shared between mammary and liver tissues.

Like chromatin state, which can be active or silent, genes can be either expressed or unexpressed. Considering all expressed and unexpressed transcripts in the two tissues, expression status was shared 85.7% of the time. However, of the 4305 genes expressed in at least one of the two tissues, only 48% were expressed in both. Thus, the shared transcriptional state between the two tissues was mainly due to the fact that most genes (72% in the Atlas data) were not expressed in either tissue. On a genome-wide scale, the epigenome and transcriptomes were similar between mammary and liver tissue, mainly due to shared silencing of gene expression.

To determine whether gene neighborhoods were consistent with chromatin domain boundaries, we reviewed all possible adjacent gene pairs for their neighborhood (TNS) and chromatin domain scores (DS). Pairs of genes were classified as active, silent, concordant (not active or silent in mammary tissue relative to liver), or discordant (one active and one silent). By this classification, 2.27% of gene pairs were uniquely active in the mammary gland relative to the liver, 4.37% were uniquely silent, 90.78% were concordant between mammary gland and liver, and 2.56% were discordant. On average, the TNS of active pairs was higher than silent, concordant, or discordant pairs (Wilcox test, p = 0.006494, p = 0.01189, p = 0.02791). The average TNS was not significantly different among silent, concordant, and discordant pairs. These results suggest, as expected, that genes sharing active chromatin domains are more likely to be coordinately expressed together.

### Mammary gene neighborhoods are primarily transcriptionally suppressed during lactation

**Figure 5:**
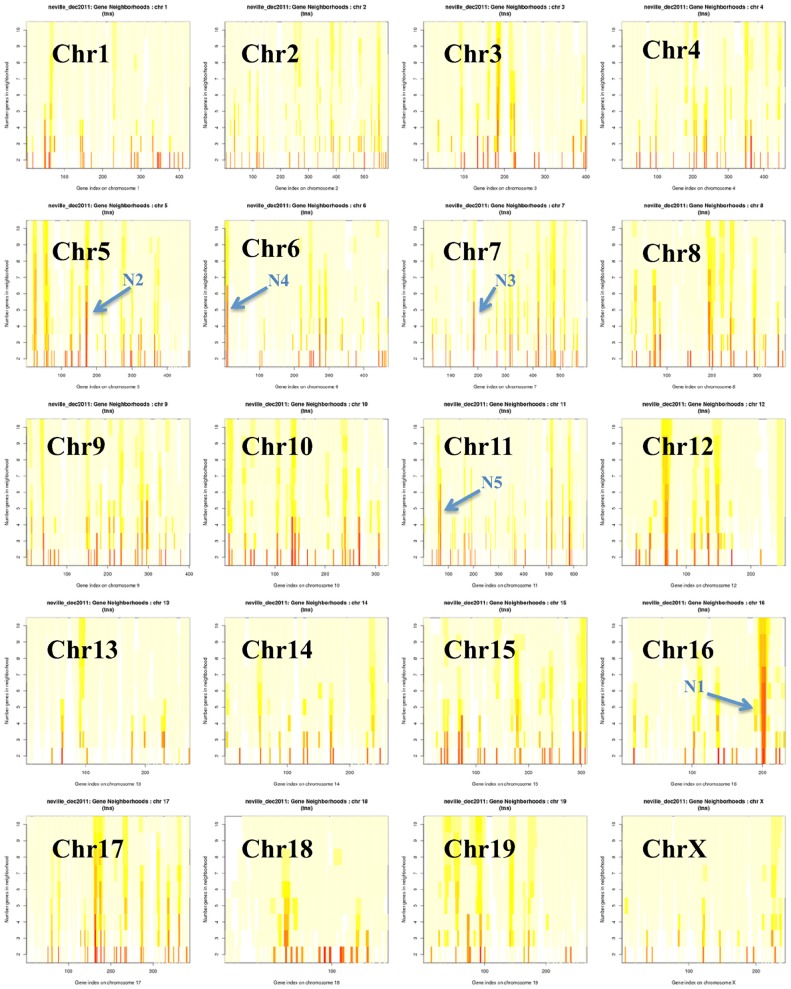
Total Neighborhood Scores (TNSs) plotted across all window sizes on all chromosomes. TNSs were computed using the Mammary data set with window sizes from 2 to 10 genes. The *x*-axis represents the gene index that is the order in which the genes appear on the chromosome. The *y*-axis represents the window size from 2 to 10 genes. This birds-eye view shows all 20 chromosomes at a glance, with the five largest high-scoring neighborhoods (Table 1) annotated as N1–N5. TNSs are indicated by color: Red, 0.6–1; Orange, 0.4–0.6; bright yellow, 0.1–0.3; light yellow, <0.1.

The “Mammary” data set (Methods) included four biological replicates at each of 10 time points, enabling a more sophisticated definition of gene neighborhoods that relied on correlates of gene expression across many conditions. Given the “Mammary” data set, we scored all possible gene neighborhoods using G-NEST [3]. For each putative neighborhood, G-NEST combines gene expression and synteny information to determine a Total Neighborhood Score (TNS) that ranges from “0” (not a neighborhood) to “1” (definitive neighborhood). A genome-wide overview of TNSs simultaneously computed across putative neighborhoods 2–10 genes in length suggested that gene neighborhoods of relevance to mammary biology were present on all chromosomes (Figure 5).

To evaluate the different characteristics of mammary gene neighborhoods, we ranked putative neighborhoods by their TNS and number of genes. The largest neighborhoods (greatest number of genes) with the highest TNS scores are listed in Table 1. Table 1 lists only genes that were probed on the microarray. The actual neighborhood may have contained additional genes. However, interleaving un-probed genes are not listed here due to the fact that they might not be part of the neighborhood. Note that even among the short list of large, top-scoring neighborhoods (Table 1), sizes ranged from 33KB to over 1 MB.

**Table 1:** Largest high-scoring gene neighborhoods in mouse mammary gland.^a^

Label	Neighborhood Score (TNS)	Probed Genes	Location	Size
N1	0.69	Mrap, Gcfc1, Ifnar2, Il10rb, Ifnar1, Ifngr2	chr16: 90738568–91565414	6 genes, 826 KB
N2	0.70	Csn1s1, Csn2, Csn1s2a, Csn1s2b, Csn3	chr5: 88095232–88361557	5 genes, 267 KB
N3	0.66	Gpi1, Lsm14a, Pepd, Cebpg, Cebpa	chr7: 34987148–35906945	5 genes, 920 KB
N4	0.64	Col1a2, Sgce, Pon1, Pon3, Pon2	chr6: 4455696–5248373	5 genes, 793 KB
N5	0.64	Ccdc88a, Mtif2, Rtn4, Spnb2, Acyp2	chr11: 29273774–30549402	5 genes, 1,276 KB
N6	0.79	Col6a2, Col6a1, Slc19a1, Col18a1	chr10: 76058506–76629246	4 genes, 570.7KB
N7	0.74	Ict1, Hn1, Nup85, Mrps7	chr11: 115265079– 115468679	4 genes, 203.6KB
N8	0.72	Psmb9, Tap1, Psmb8, Tap2	chr17: 34320077–34353264	4 genes, 33.2KB
N9	0.67	Snapin, Fop, S100a1, S100a13	chr3: 90291947–90328503	4 genes, 36.5KB
N10	0.67	1110002B05Rik, Snx6, 2700097O09Rik, Psma6	chr12: 55746360–56519436	4 genes, 773.1KB
N11	0.67	Derl1, Zhx1, D15Ertd621e, Ndufb9	chr15: 57701056–58771044	4 genes, 1,070KB
N12	0.62	Prdx2, Junb, Asna1, 2310036O22Rik	chr8: 87493598–87554184	4 genes, 60.6 KB
N13	0.61	Cct2, Lyz2, Lyz1, Mdm2	chr10: 116488058– 117147814	4 genes, 659.8KB

a Sorted by Neighborhood Size (in Probed Genes), then by TNS.

**Figure 6:**
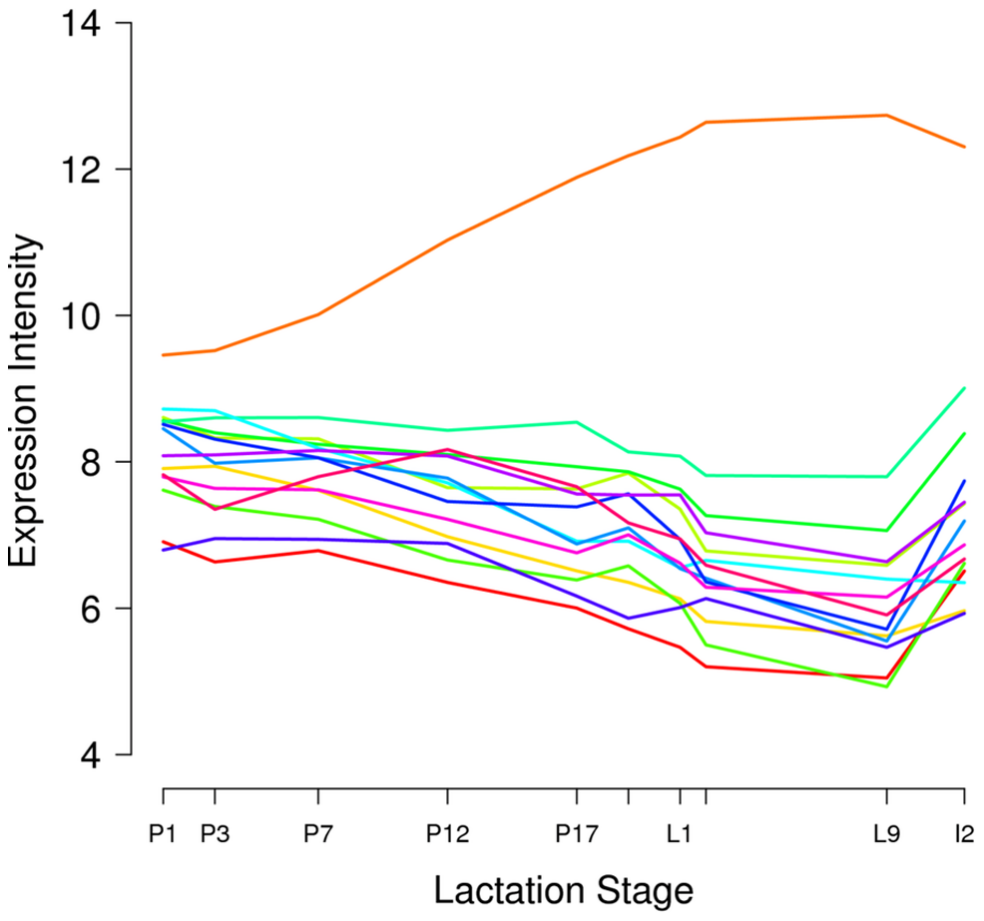
Average gene expression trajectories of largest high-scoring gene neighborhoods in mouse mammary gland. For each of the 14 unique gene neighborhoods with four or more genes in Table 1, the gene expression intensity at each time point was averaged across genes in the neighborhood and plotted. Each color represents the trajectory of a different neighborhood. The “orange” line represents the casein gene neighborhood (Csn1s1, Csn2, Csn1s2a, Csn1s2b, Csn3).

Next, for each of the top-scoring large neighborhoods (Table 1), we computed an “average” expression profile by averaging the gene expression intensity across all replicates and all genes in the neighborhood at each time point. These neighborhood expression trajectories (Figure 6) showed that nearly all high-scoring large mammary neighborhoods contained genes that were suppressed during lactation relative to other developmental states (early or late pregnancy, involution). Only one neighborhood – the casein milk protein genes – was highly expressed during lactation.

To determine whether genes expressed or differentially regulated during lactation were more likely to be members of gene neighborhoods, we computed the highest TNS associated with each gene for all of its putative neighborhoods and compared these best TNS values with the expression status of the genes. We specifically compared the transcriptional state in lactation with that of late pregnancy because cell populations were not changing in the mouse mammary gland during this time. As expected, genes expressed during late pregnancy or lactation were associated with higher TNS scores than genes not expressed during these states (Wilcox, p-value <2.2e-16; KS, p-value <2.2e-16). In other words, genes expressed in late pregnancy or lactation were enriched in neighborhoods. Interestingly, genes that are down-regulated during lactation relative to late pregnancy were more likely to have high TNS scores than genes that were up-regulated (W, p = 7.995e-09; KS, p = 8.563e-09) or not significantly regulated (W, p = 1.843e-12; KS, p = 2.213e-12). Thus, genes that were down-regulated during lactation were more likely to be in gene neighborhoods than those that were up-regulated.

### Mammary gene neighborhoods were primarily epigenetically silenced during lactation; uniquely active genes were primarily isolated

**Figure 7:**
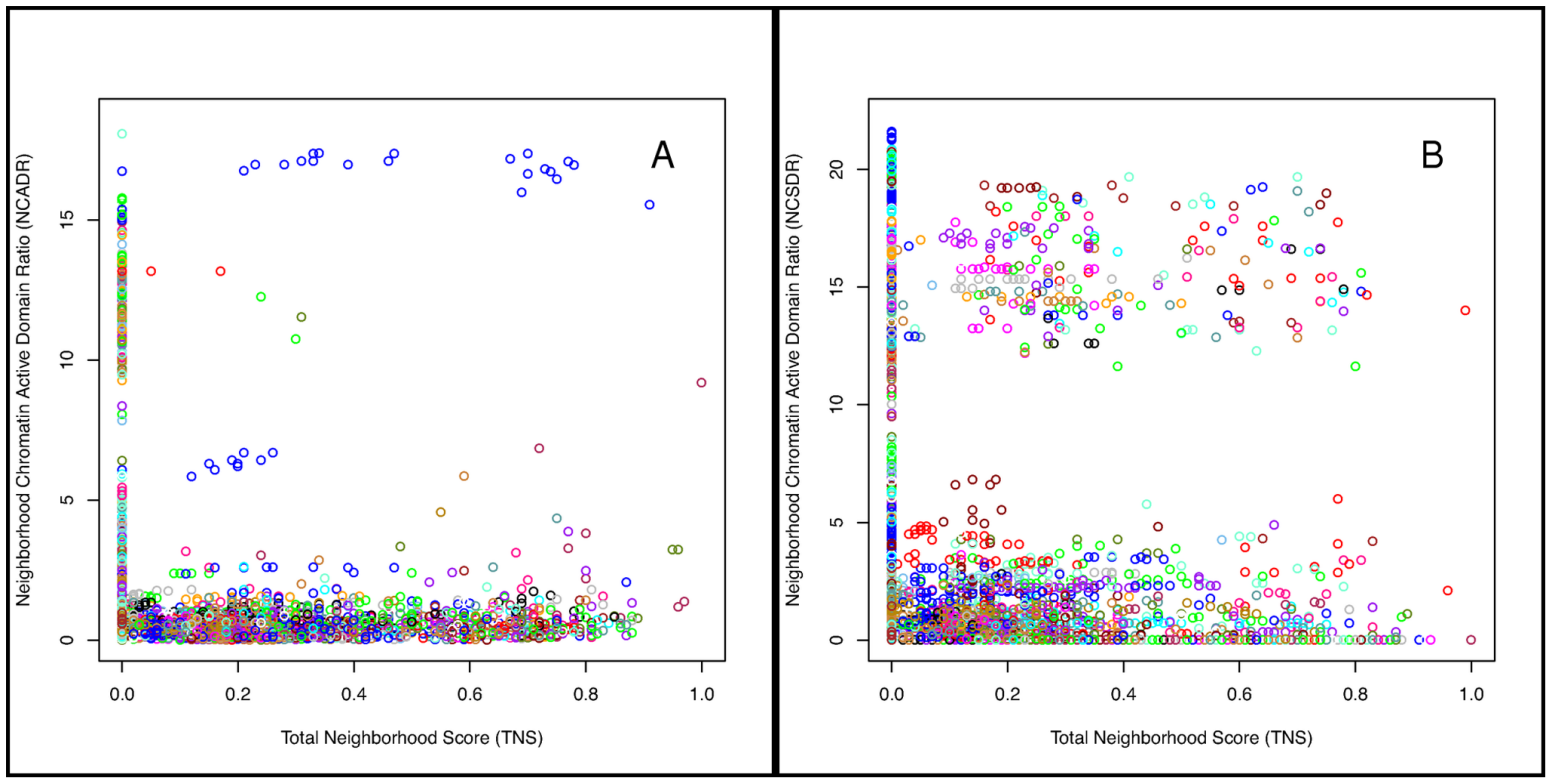
Neighborhood-level chromatin domains. (A–B). Each point in the figure corresponds to a putative gene neighborhood. The *x*-axis shows its Total Neighborhood Score (TNS) and the *y*-axis shows the mammary-to-liver Neighorhood Chromatin (A) Active or (B) Silent Domain Ratio. Points are colored according to the chromosome on which the putative neighborhood resides. (A) The many blue points with high NCADR and high TNS correspond to the casein neighborhood (N2 in Table 1) and its derivatives.

Given that most gene neighborhoods in the mammary gland are transcriptionally suppressed during lactation, we asked whether this observation was also reflected in the chromatin state of those neighborhoods. TNS were computed for all possible gene neighborhoods using the “Mammary” gene expression set. For each putative neighborhood, we also computed a Neighborhood Mammary/Liver Chromatin Active Domain Ratio (NCADR) and Neighborhood Mammary/Liver Chromatin Silence Domain Ratio (NCSDR), for neighborhood-wide active and silenced domains, respectively (Methods). The TNS, NCADR, and NCSDR were plotted for every putative neighborhood (Figure 7).

All putative neighborhoods with the highest NCADR scores were derivatives of the casein neighborhood (blue points, Figure 7). On the other hand, many more unique gene neighborhoods appeared to be associated with closed chromatin in the mammary gland, relative to liver tissue (Figure 7). Of the 21 mouse chromosomes, 16 contained at least one highly silenced gene neighborhood (NCSDR >10, TNS >0.4). In contrast, only one neighborhood – the casein genes – was highly active (NCADR >10). In summary, most neighborhoods with a common chromatin state unique to the mammary gland (relative to the liver) appeared to be associated with closed chromatin. In other words, these neighborhoods of genes were active in the liver, but not the lactating mammary gland. The closed chromatin state in the mammary gland was consistent with gene expression data, which showed more down-regulation of genes during lactation relative to pregnancy.

While comparing neighborhood and chromatin domain scores, it became obvious that many chromatin domains may span only a single gene. This was apparent in the cluster of points along the y-axis where TNS  = 0 in Figure 7. We therefore re-examined the CADR, CSDR, and TNS scores for individual genes based on the “Mammary” data set. Mammary “active” genes – those with histone marks associated with more open chromatin relative in the mammary gland to liver (CADR >8) – had lower neighborhood scores compared with other genes (Wilcox, p = 0.0010; KS, p = 0.0085). Mammary “silenced” genes (CSDR >8) had neighborhood scores that were not significantly different compared with other genes (Wilcox, p = 0.6689; KS, p = 0.9915). In other words, uniquely “active” mammary genes were more likely to be isolated while uniquely “silenced” genes occurred in neighborhoods with equal likelihood as other genes. This may be due to the fact that K27me3 marks usually covered more than one gene.

A limitation of the NCADR and NCSDR scores is that they have a “shadow” effect. For example, if genes A, B, and C are all associated with high active domain scores and gene D has a lower score, the putative neighborhood of A, B, C, D will still score highly, even though gene D is not really part of the neighborhood. To circumvent this problem, we tried an alternate approach to identify gene neighborhoods within active chromatin domains by comparing each gene's DS with its best TNS (Methods). On average, genes with high DS (active domain) had a lower TNS score compared with other genes. Genes with a negative DS (silent domain) did not have a significantly different TNS compared with other genes. These results confirmed that uniquely active genes were more likely to be isolated. In other words, those genes with open chromatin status unique to the mammary gland, relative to the liver, were less likely to be in gene neighborhoods.

### Epigenetically uniquely active domains highlight genes and gene neighborhoods important to lactation

**Figure 8:**
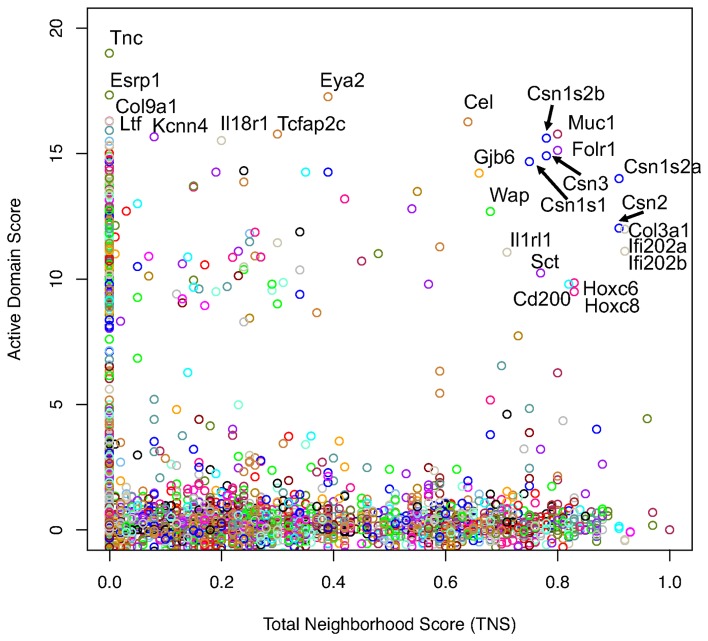
Genes and gene neighborhoods uniquely active in the mammary gland. Each point corresponds to a single gene. The *x*-axis is the best Total Neighborhood Score (TNS) associated with each gene and the *y*-axis is the gene's Domain Score (DS). Points with the same color indicate genes on the same chromosome. Genes with highest associated TNS and/or DS are annotated.

To find genes of unique importance to lactation, we examined uniquely active genes (DS >2), both within and outside of gene neighborhoods. Figure 8 displays a plot of the DS (for DS >0) compared with the best TNS for each gene. Points with high TNS and/or DS were annotated with the associated gene symbol. As expected, the casein genes were present among high DS and TNS, but a number of other important milk proteins, such as whey acidic protein (Wap), mucin 1 (Muc1), and bile salt stimulated lipase (Cel), were as well. Genes with high DS, but isolated (TNS  = 0) also included well-known milk proteins such as lactoferrin (Ltf) (Table 2).

**Table 2:** Top ten isolated genes (TNS = 0) uniquely active in the mammary gland.^a^

Gene Symbol	Ensembl Transcript ID	ADS	Description
Tnc	ENSMUST00000070019	19	tenascin C
Esrp1	ENSMUST00000108313	17.33	epithelial splicing regulatory protein 1
Sox10	ENSMUST00000040019	16.28	SRY-box containing gene 10
Col9a1	ENSMUST00000054588	16.28	collagen type IX, alpha 1
Ltf	ENSMUST00000035077	15.93	lactotransferrin
Actn2	ENSMUST00000064204	15.49	actinin alpha 2
Serpinb5	ENSMUST00000086701	15.32	serine (or cysteine) peptidase inhibitor, clade B, member 5
Spint1	ENSMUST00000028783	15.29	serine protease inhibitor Kunitz type 1
Tspan8	ENSMUST00000080630	15	tetraspanin 8
Krt19	ENSMUST00000007317	14.89	keratin 19

a ADS, Active Domain Score.

Of the 527 “active” genes (DS ≥2), there were 53 in neighborhoods (TNS >0.4), 369 not in neighborhoods (TNS <0.01), and 105 indeterminate (0.01< TNS <0.4). The list of 53 “active” neighborhood genes (best TNS >0.4, DS ≥2) was reviewed manually and distilled to just four neighborhoods with all members in uniquely “active” domains (Table 3). Most of the 53 genes were neighbors with other genes that were not uniquely active in the mammary gland, but rather, had shared chromatin status with the liver. The top genes and gene neighborhoods that were uniquely active in the mammary gland, relative to the liver (Tables 2 and 3), contained many genes known to be important to lactation.

**Table 3:** Gene neighborhoods (TNS >0.4) uniquely active in the mammary gland.^a^

Gene Symbols	Ensembl Transcript ID	Chr	ADS	Gene Descriptions
Csn1s2b	ENSMUST00000072539	5	15.61	casein alpha s2-like B
Csn1s2a	ENSMUST00000076379	5	14.00	casein alpha s2-like A
Csn2	ENSMUST00000082370	5	12.03	casein beta
Csn1s1	ENSMUST00000094641	5	14.68	casein alpha s1
Csn3	ENSMUST00000001667	5	14.90	casein kappa
Muc1	ENSMUST00000041142	3	15.77	mucin 1
Trim46	ENSMUST00000107464	3	6.25	tripartite motif-containing 46
Elf5	ENSMUST00000028609	2	6.32	E74-like factor 5
Ehf	ENSMUST00000111172	2	5.45	ets homologous factor
Sct	ENSMUST00000046156	7	10.24	secretin
Drd4	ENSMUST00000026569	7	3.21	dopamine receptor 4

a Chr, Chromosome; ADS, Active Domain Score.

Given that gene neighborhoods are, by definition, highly conserved, we hypothesized that neighborhoods would be enriched with regulatory nodes. None of the 11 uniquely active genes within neighborhoods (Table 3) were network hubs, defined here as having 10 or more known protein interactions in a mouse protein interaction database (lgsun.grc.nia.nih.gov). Meanwhile, three of the top ten isolated genes listed in Table 2– Sox10, Actn2, and Krt19– were hubs. Thus, uniquely active genes within neighborhoods were less likely than expected to be regulatory nodes (chi square, p<0.0001). Clearly, neighborhood membership was not associated with increased regulatory network connections.

## Discussion

Biology continually reuses components to create new cells, tissues, and organisms that function in new and unique ways. Despite the fact that the mammary gland only recently evolved relative to more ancient organ systems, gene neighborhoods that contribute to the mammary transcriptome have a similar size and distribution compared with other tissues, probably due to the fact that these neighborhoods are *shared* with other tissues. In other words, neighborhoods of genes that are co-expressed in the mammary gland are also co-expressed in other pre-existing tissues.

In this study we showed histone marks to be remarkably self-consistent as well as consistent with known biological functions. As expected based on recent work with other tissues [19]–[21], the use of multiple histone marks to determine chromatin state was more effective than any single histone mark alone. Combining the chromatin state with the comparative analysis of a tissue with a divergent control tissue was essential to the identification of features of interest. Patterns of histone mark ChIP-Seq peaks are complex, yielding few firm and fast rules. On the other hand, these patterns appear to be largely conserved across tissues and other biological states such that the use of one or more control tissues enables the rapid identification of divergent peak patterns.

Interestingly, two tissues that are among the most divergent in gene expression – the mammary gland and the liver – have a remarkably similar epigenome, based on the three histone marks used in this study. More than 80% of the epigenomes and transcriptomes were shared between the two tissues. Their similarity is due to the fact that most genes are not expressed in either tissue. In principle, with each functional differentiation of cells, their transcriptional repertoire narrows and more genes are silenced. Our results suggest that the silencing is largely shared and what distinguishes mammary gland from liver tissue is primarily the result of fairly small tissue-specific repertoires of expressed genes.

We observed several notable features of gene expression during lactation. First, genes in most neighborhoods were suppressed during lactation as reflected in their expression levels and their location in regions of silenced chromatin. Second, neighborhoods of genes uniquely active in the lactating mammary gland as compared with other tissues were extremely rare. Furthermore, the few genes within uniquely epigenetically active mammary neighborhoods were not regulatory nodes, although some are vital to lactation and yield well-known milk proteins. On the contrary, genes uniquely active in mammary tissue compared with liver tissue were depleted in neighborhoods and were more likely regulatory nodes. This genomic distribution – of isolated genes – is similar to what we previously described for “essential” genes, which are also rarely located in neighborhoods [3], even though the mammary-specific genes are not categorized as essential per se. This finding suggests that genes essential to an organ's processes may share some properties, at least in terms of their genomic distribution, with truly essential genes – those essential to an organism's survival and/or reproduction. Thus, gene neighborhoods may well be comprised of genes with greater tolerance for variation in expression and therefore contribute less to a cell or tissue's uniqueness.

The repressive signature of most neighborhoods suggests that a potential advantage of the neighborhood organization is in the collective repression of groups of genes. This is consistent with the concept that repressive chromatin state “spreads” and covers multiple genes. K27me3 is found in BLOCs that cover more than one gene [7]. Similar patterns of collective repression were observed with K9me2, another silencing mark [22].

Recent studies suggested that co-repression of gene neighbors also might relate to the large-scale nuclear organization of chromatin [23]. We previously observed that mammary epithelial cells in lactating tissue have a different nuclear distribution of heterochromatin (closed chromatin) than in less differentiated states (virgin and pregnancy) and lactation-associated genes change their location within the nucleus upon stimulation of gene expression [24]. Furthermore, developmental stage seems to influence 3D chromatin organization of lactation associated genes and regulatory elements [25]. Global transitions of chromatin states associated with development and differentiation are related to chromatin and nuclear architecture [23]. Clearly, elucidation of the 3D chromatin organization of genes is needed to fully understand the regulation of gene expression. Technologies for genome-wide mapping of chromatin architecture have been developed, such as Hi-C [26], [27] and are being extended to the sub-megabase scale [28]. Such technologies could be employed in future studies.

In a prior study, we found widespread down-regulation of transcription in the mammary gland during lactation, relative to pregnancy [29]. By what mechanism is such widespread suppression of transcription achieved? The data presented in this paper suggested relatively similar amounts of closed chromatin in the mammary gland compared with the liver. In fact, when the chromatin silencing ratio of the two tissues was computed based on the H3K27me3 marks (log (CSDR)) for every gene, the genome-wide median was zero, suggesting a near equal amount of closed chromatin in the two tissues. The majority of these regions harbor the same genes that are silenced in both tissues, as they do not contribute to the expression repertoire that defines the tissue identity and function. A future study explicitly comparing the chromatin state of the mammary gland during pregnancy and lactation is needed to determine to what extent epigenetic modification of the genome resulting in chromatin silencing could be responsible for the down regulation of the large number of genes during the pregnancy-lactation transition.

The well-known casein gene neighborhood, which gives rise to the most abundant milk protein genes, was the motivation for seeking additional gene neighborhoods of importance to the lactating mammary gland. With a few exceptions (Table 3), this approach appeared to be of limited utility. Instead, we found that gene neighborhoods were not important in the context of what genes are turned “on,” but rather, what genes are turned “off.” The data represented here suggested that coordinated regulation via chromatin silencing might be an important contributor to the maintenance of gene neighborhoods during evolution. Organization of genes into neighborhoods enables the silencing at once of a large set of genes not needed to define further developmental or functional differentiation stages of a cell by changing chromatin state and nuclear localization.

There are several limitations to this study. First, all analyses were based on microarray data, which are known to be limited by the probes on the array. We mitigated this effect by disregarding unprobed genes when defining gene neighborhoods, although our results were nonetheless underpowered. Second, the ChIP-Seq data were generated using a strain of mouse that differed from the strain on which the gene expression data were based. Given that epigenetic patterns within a given tissue are largely conserved between individuals of the same species [30]–[31] and our analyses compared data across many genomic regions, we expected this discrepancy to have only a small impact on our results. Transcriptomic patterns within a given tissue are highly correlated between strains of the same species [32]–[34]. While the mammary transcriptomes of the two strains have never been directly compared, we can surmise from inter-strain studies of mouse brain, liver, and muscle tissue [32]–[34], that 1–3% of expressed genes may be significantly different between the strains. These studies further indicate that developmental stage or tissue-specific gene expression changes will be 10-fold greater than inter-strain differences. Third, with the use of the liver as a control tissue, any genes or gene neighborhoods of importance to lactation that have a shared importance with the liver, would go undetected. The results of this study suggested that comparative genomics in concert with a single transcriptome cannot reliably substitute for more advanced comparative transcriptomic studies. Future studies should incorporate the epigenomes, transcriptomes, and/or proteomes of multiple species or stages of mammary development.

## Conclusions

The genomes of all somatic cells are nearly identical and yet these same genes give rise to functions as diverse as sensory perception and milk production. Many regulatory layers of gene expression enable this diversity of function. This study presents a picture of the genome, epigenome, and transcriptome of the mammary gland during lactation relative to liver tissue. Uniquely active gene neighborhoods were extremely rare. The “default” epigenomic state appeared to be neighborhood-wide closed chromatin. This suggests that an advantage of the neighborhood organization is in the collective repression of groups of genes via a shared mechanism of chromatin repression. Mammary-specific domains of active chromatin were primarily associated with isolated genes. Like genes responsible for an organism's survival, genes essential to the mammary gland's uniqueness have similar properties: isolated from neighbors, with less tolerance for variation in expression. While this picture was elucidated using mice, we expect it to hold for other mammals such as humans and dairy cows.

## Supporting Information

Figure S1 **Calculation of the Chromatin Active Domain Ratio (CADR).** The diagram is a UCSC Genome Browser display of the SICER peaks called for the histone marks in an example locus on mouse chromosome 5, assembly mm9.
(PDF)

Figure S2 **Correlation of genomic distance with gene expression across 61 tissues.** The *x*-axis is genomic distance in Kb. The *y*-axis is average correlation. Each circle represents the mean correlation of all gene pairs within that genomic interval on the same chromosome. The red line indicates the mean correlation of gene pairs on different chromosomes.
(PDF)

Figure S3 **Gene neighborhood size (in KB) in several tissues.** The *x*-axis is the tissue and the y-axis is size of gene neighborhoods (defined here as adjacent co-expressed genes) in KB. The “box” part of each box-and-whisker shows the median gene neighborhood size while the “whiskers” denote the 5^th^ through the 95^th^ percentiles.
(PDF)

File S1 **Gzipped custom track for the UCSC Genome Browser, mouse assembly mm9, displaying** **TNS.**
(GZ) Click here for additional data file.

File S2 **Gzipped tab-delimited file reporting locations and scores for all putative gene neighborhoods.**
(GZ) Click here for additional data file.

File S3 **Gzipped tab-delimited file reporting the best TNS for each gene probed on the microarray.**
(GZ) Click here for additional data file.
