# Supplementary figures and images for: From Genes to Milk: Genomic Organization and Epigenetic Regulation of the Mammary Transcriptome
Source: PLoS One. 2013 Sep 26;8(9):e75030. doi: 10.1371/journal.pone.0075030 (PMC3784412; doi:10.1371/journal.pone.0075030)

# Neighborhood Chromatin Active Domain Ratio

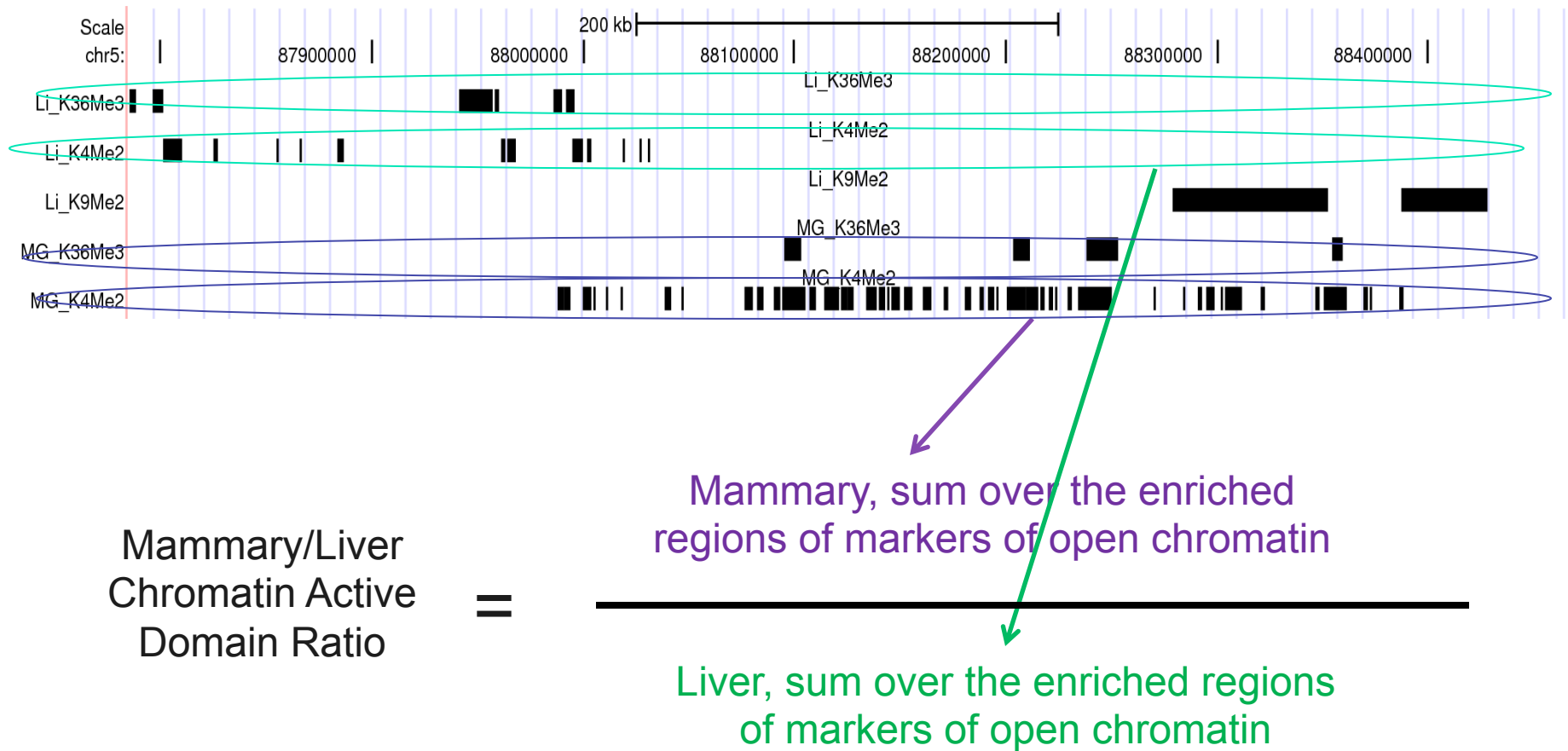

Supplement: Figure S1 — Calculation of the Chromatin Active Domain Ratio (CADR). The diagram is a UCSC Genome Browser display of the SICER peaks called for the histone marks in an example locus on mouse chromosome 5, assembly mm9. (PDF) [file pone.0075030.s001.pdf]

# All-tissue data: correlation by genomic distance

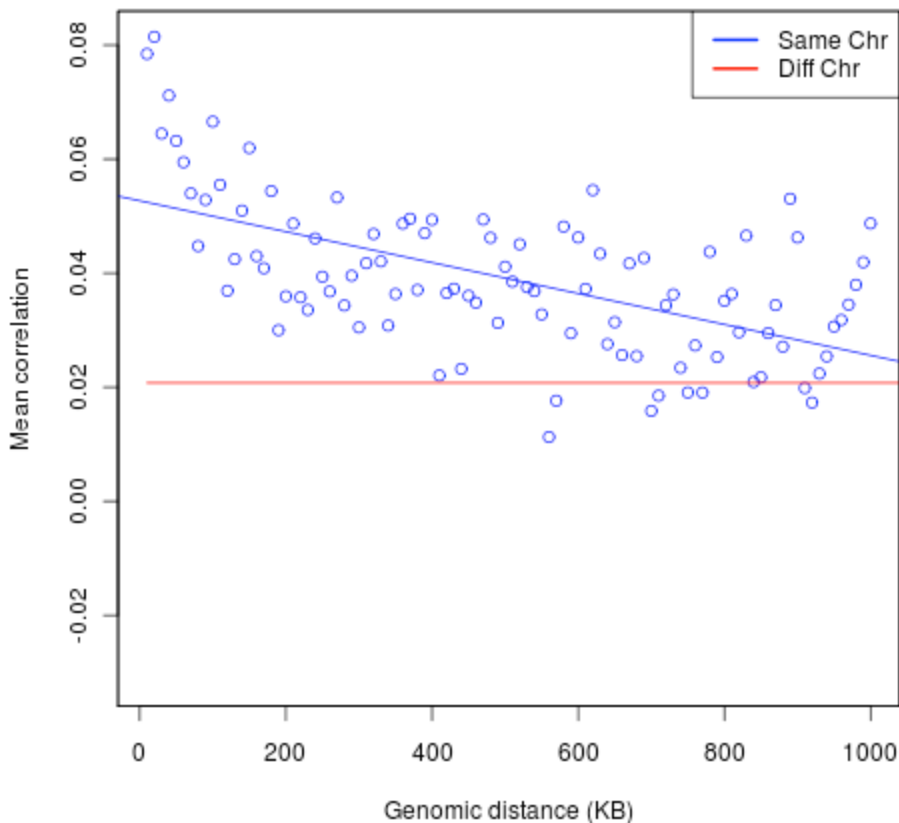

Supplement: Figure S2 — Correlation of genomic distance with gene expression across 61 tissues. The x-axis is genomic distance in Kb. The y-axis is average correlation. Each circle represents the mean correlation of all gene pairs within that genomic interval on the same chromosome. The red line indicates the mean correlation of gene pairs on different chromosomes. (PDF) [file pone.0075030.s002.pdf]

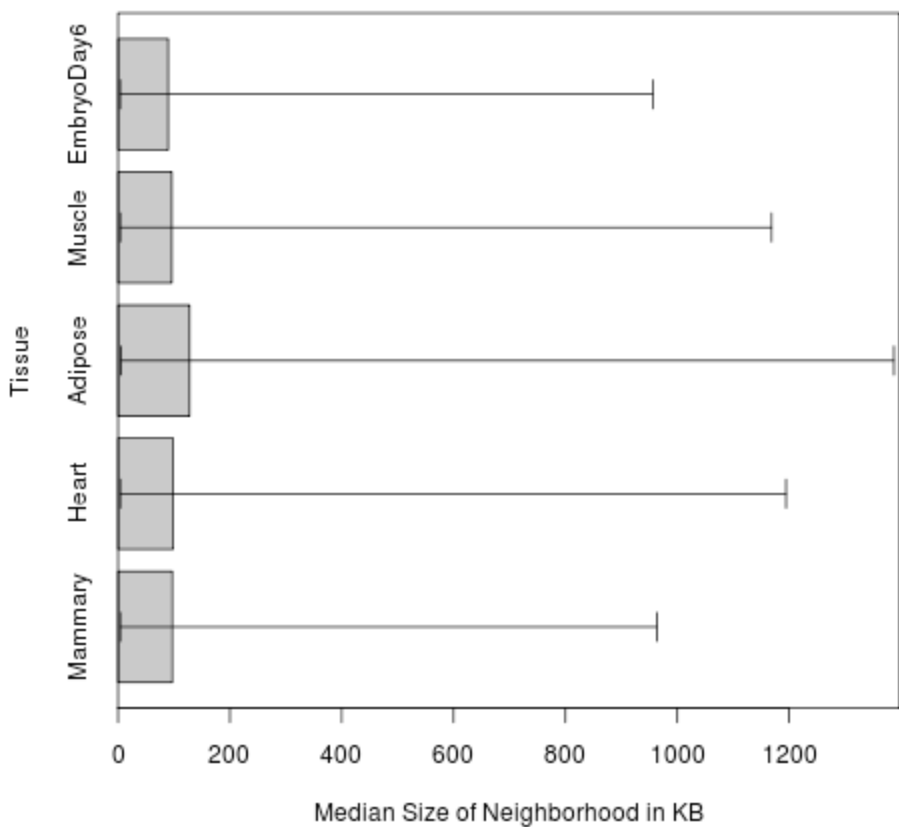

Supplement: Figure S3 — Gene neighborhood size (in KB) in several tissues. The x-axis is the tissue and the y-axis is size of gene neighborhoods (defined here as adjacent co-expressed genes) in KB. The “box” part of each box-and-whisker shows the median gene neighborhood size while the “whiskers” denote the 5th through the 95th percentiles. (PDF) [file pone.0075030.s003.pdf]
